# Are Lockdown Measures Effective Against COVID-19?

**DOI:** 10.3389/fpubh.2020.549692

**Published:** 2020-10-22

**Authors:** Samer Kharroubi, Fatima Saleh

**Affiliations:** ^1^Department of Nutrition and Food Sciences, Faculty of Agricultural and Food Sciences, American University of Beirut, Beirut, Lebanon; ^2^Department of Medical Laboratory Sciences, Faculty of Health Sciences, Beirut Arab University, Beirut, Lebanon

**Keywords:** COVID-19, lockdown measures, Poisson regression model, modeling, Lebanon

## Abstract

As the Coronavirus Disease 2019 (COVID-19) pandemic progresses, countries around the world are increasingly implementing a range of responses that are intended to help prevent the transmission of this disease. In the absence of a COVID-19 vaccine, we assess the potential role of containment measures to suppress the virus transmission, thereby slowing down the growth rate of cases and rapidly reducing case incidence. The aim of this study is to show that country lockdown has a critical and significant impact on the pandemic. This is explored using real time incidence data in Lebanon. We analyze COVID-19 cases in Lebanon before and after lockdown measures have been implemented. The findings show that the nationwide lockdown was effective in reducing cases and has been successful in, so far, containing the virus. This study could be an evidence-based call to continue with the lockdown measures, based on real time incidence data. Further research is encouraged.

## Introduction

Many authors have tried to predict the incidence of COVID-19 in China (1). The outbreak, nonetheless, has rapidly accelerated outside China and was declared by the World Health Organization (WHO) as a global pandemic on March 11, 2020 (2). Thus, serious debates were raised on ways to react to the transmission of this disease. China was the first to implement a complete lockdown of Wuhan and at least 16 other cities by the end of January to try to contain the causal virus (SARS-CoV-2), then the world followed, which can now be described as the largest quarantine in human history. However, an alternative approach was adopted by many countries including the UK and the US that is to achieve herd immunity. Herd immunity refers to having as many lower risk people infected as possible and thus become immune while minimizing the exposure of people who are vulnerable (3, 4).

The Lebanese Ministry of Public Health, along with its Mediterranean peers, has been explicitly attempting to limit the spread of the virus and, hopefully, eradicate it. Hence, the Lebanese government has adopted one of the strictest lockdowns in the Middle East and North Arica (MENA) region even before a single death was reported, and has isolated infections to keep the disease at bay. As a matter of fact, the Lebanese government was trying to combat COVID-19 amid its worst financial and economic crisis since the Lebanese civil war and at a time of wide spread protests raging across the country demanding political and economic reforms.

In this study we aim to show, with real time incidence data, that the country lockdown imposed by the Lebanese government will have a critical and significant impact on reducing the spread of the virus and eventually containing it. More specifically, we present results of an analysis of COVID-19 cases in Lebanon pre- and post-lockdown measures. The data show that post-lockdown daily cases decreased, thereby, demonstrating that the lockdown measures have been successful in, so far, containing the disease. To the best of our knowledge, this study would be the first in the Middle East to analyze and predict the spread of COVID-19 before and after containment measures such as lockdowns have been implemented, and would therefore be of benefit to neighboring countries until similar studies are conducted in the region.

## Methods and Results

On February 21, Lebanon, a small country in the Middle East, reported the first case of COVID-19 for a 45-year-old woman traveling from Iran. With a deepening economic crisis and lack of robust health system, the arrival of the coronavirus was unwelcome and particularly alarming. However, the government imposed strict measures in response to COVID-19 with a strategy to flatten the curve while increasing the capacity of the health care system to adequately respond to this pandemic. A week after the first case was confirmed, the Lebanese government announced the closure of all educational institutions. On March 6, the Ministry of Public Health declared that Lebanon is no longer in COVID-19 containment stage and thus urged the public to avoid gatherings followed by closure of all theaters, gyms, restaurants and pubs. On the 15th of March, the Lebanese government declared a state of health emergency and adopted sweeping measures, including full lockdown, shutting down airport, imposing travel restrictions and completely sealing the borders, as part of the country's efforts to combat the spread of COVID-19. All of these measures have the potential to suppress the virus transmission from one person to another, thereby, slowing down the growth rate of cases and rapidly reducing case incidence. It is perhaps worth commenting that containment measures implemented in Lebanon to date are in line with WHO's recommendations, and are similar to those implemented by other countries. New Zealand, for example, adopted strict lockdown measures before a single death was reported (March 23) with the aim to eradicate the virus.

In terms of numbers, as of March 24, a total of 304 cases of COVID-19 has been declared in Lebanon (5). At the country level, the incidence rate was equivalent to 39 cases per 1 million of the Lebanese population. In comparison to other countries, this was in line with New Zealand (38 cases/1 million of New Zealanders), which is widely regarded as having done a good job of managing the spread of COVID-19 and has a slightly smaller population−5.3 million compared with Lebanon's 6 million. However, the incidence rate seemed to be well below countries where the outbreak has started around the same time but has been substantial (e.g., Italy: 1,057 cases/1 million; Spain: 708 cases/1 million), yet higher than other countries in the Middle East (Jordan: 11 cases/1 million; Saudi Arabia: 16 cases/1 million) (6). Over the last 2 weeks (March 21–April 3), the growth of new cases appeared to be slowing down (5). This could give rise to critical and highly significant questions: Are containment measures working? Does this recent data suggest that the measures are effective and starting to show an impact?

To answer these questions, the incidence of COVID-19 in Lebanon was predicted by applying a Poisson regression model using data on the daily number of new COVID-19 occurrences since 21st of March. In statistics, Poisson regression is a generalized linear model form of regression analysis used to model count data as a function of a set of predictor variables. A model of this kind has been extensively used both in human and in veterinary epidemiology to investigate the incidence and mortality of many different infectious diseases, for instance malaria (7), SARS (8), MRSA (9), Ebola (10), and mastitis (11). Herein, we propose to adapt this model to the COVID-19 contagion in Lebanon. All analyses were carried out using the statistical software R (12).

**Figure 1:**
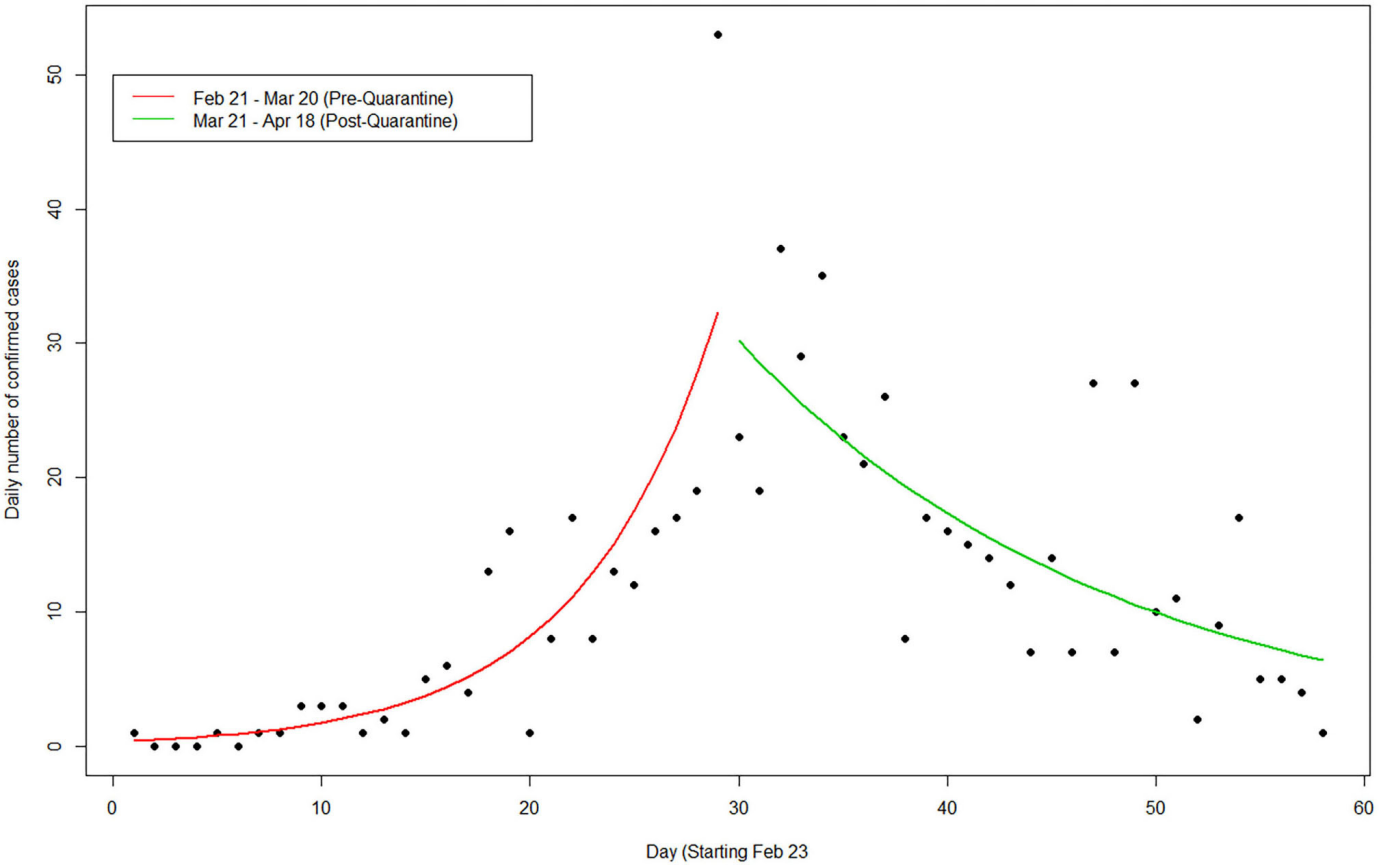
Lebanon confirmed cases rate based on a Poisson regression model.

The Poisson regression model was applied pre- and post the nationwide lockdown enforced by the Lebanese government. The pre-quarantine period started from February 21 (when the first case of COVID-19 was reported) until March 20 (a week after wide-reaching lockdown measures were announced). The post-quarantine included the period from March 21–April 18, which showed the recent reduction in the number of new cases being recorded each day. The resulting predicted mean occurrences using the Poisson model pre- and post-quarantine along with the actual occurrences are depicted in Figure 1. As can be seen from the plot, the Poisson model (red line) showed a significant growth trajectory in the number of cases during the pre-quarantine period. Figure 1 also revealed an inflection point where the growth of cases has slowed down and the number of cases has declined significantly during the post-quarantine period (green line). This supports the claim that the containment measures implemented were effective at this stage in containing the outbreak.

## Discussion

As the COVID-19 pandemic progresses, countries worldwide are increasingly implementing a broad range of responses that are intended to help preventing the spread of this disease. Our results demonstrate that it will be extremely important to continue to enforce and/or adhere to containment measures implemented in Lebanon. These measures have shown to have a critical and significant impact and appeared to be crucially effective, given the growth of new cases has slowed down significantly as well as the overall number of new cases has potentially decreased over the past month.

This has been shown in other countries that introduced early, and even delayed, locked down. New Zealand, for example, was unique among western countries in adopting strict lockdown measures to eradicate the virus and the early results were promising. The rate of new infections has decreased to the lowest in a matter of weeks, and the death toll was one of the lowest among developed nations. Other countries resorted to such measures only after fatalities soared. Nations including the UK and the US opted for such mitigation and suppression efforts after they found themselves overwhelmed by cases. More specifically, when the UK announced its lockdown on March 23, it had 6,650 Covid-19 cases and 335 people had already died (2).

However, when assessing how countries approached the coronavirus pandemic, Lebanon, being a small country in the Middle East, is sure to stand out given the crumbling economy, political chaos and the overstretched and fragmented healthcare system. The crisis in Syria has forced millions of Syrians to flee their country and find refuge outside Syria. Lebanon, in particular, hosts an estimate of 1.5 million Syrian refugees which is the largest number of refugees per capita. Now several years into the Syrian crisis, the Lebanese healthcare system is already vastly strained and overstretched. Another challenge facing the healthcare sector is the shortage of medical supplies (for example, mechanical ventilators and protective gear) necessary to deal with the COVID-19 pandemic given that all supplies are imported in US dollars and there is currently extreme dollar shortage in the country along with the liquidity crunch and the de facto devaluation of local currency.

Further significant challenge worth mentioning is that enforcement of a lockdown without socio-economic support is not sustainable. In Lebanon, a high proportion of the society already lives under the poverty line and the country's recent economic turmoil adds insult to injury. Economic conditions continue to worsen creating a level of hardship that many families found themselves unable to cope with. Governmental and non-governmental organizations as well as international agencies have been discussing different types of social and economic supports. However, these have not been materialized sufficiently yet. Thus, as a matter of urgency, extensive coordination, support, and more engagement of local communities and municipalities must be employed to ensure people continue to adhere to containment measures.

Despite all the above challenges, the early response of the Ministry of Public Health in handling the pandemic, Lebanon's relatively young population and the crucial role the media played in launching “stay at home” campaigns and spreading fear and panic amongst Lebanese, have all helped in the fight against COVID-19. At the region-level, the governmental decision, in an unprecedented move, to shut down its land borders, airport, and seaports at an early stage of the pandemic has played a significant role in controlling the spread of the virus as well as maintaining the lowest rate of infection among the surrounding countries and in the region.

We believe this study could be an evidence-based call to continue with the lockdown measures, based on real time incidence data. We predict that the country lockdown will have an impact on the pandemic, under the assumption that people continue to adhere to containment measures. These measures need to remain in place for as much of the epidemic period as possible. We stress that strict enforcement of these measures should be coupled with increased testing (and ensure that testing is available for free), contact tracing and various socio-economic support to citizens to ensure and enhance compliance. We also emphasize it is not certain that these containment measures will remain effective in the long term. Future decisions on time and length of containment to relax measures will need to be informed by ongoing surveillance. Perhaps, close monitoring of the situation in Lebanon in the coming weeks will possibly help shape strategies in other countries.

To this end, this small middle-eastern country, swinging on the edge of economic ruin and political chaos, has somehow performed something right and timely when it comes to COVID-19.

## Author Contributions

SK participated in the data acquisition, data analysis, data interpretation, manuscript drafting, and the final review of the manuscript. FS participated in the conceptualization of the idea, the design of the methodology, data interpretation, the writing of the original manuscript draft, and the final review of the manuscript. All authors have read and given final approval of the version to be published.

## Conflict of Interest

The authors declare that the research was conducted in the absence of any commercial or financial relationships that could be construed as a potential conflict of interest.
